# Enhanced Immune Responses in Mice Induced by the c-di-GMP Adjuvanted Inactivated Vaccine for Pseudorabies Virus

**DOI:** 10.3389/fimmu.2022.845680

**Published:** 2022-03-31

**Authors:** Liting Hou, Xiaoming Yu, Yuanyuan Zhang, Luping Du, Yuanpeng Zhang, Haiwei Cheng, Qisheng Zheng, Jin Chen, Jibo Hou

**Affiliations:** ^1^National Research Center of Veterinary Biological Engineering and Technology, Jiangsu Academy of Agricultural Sciences, Nanjing, China; ^2^Institute of Veterinary Immunology and Engineering, Jiangsu Academy of Agricultural Sciences, Nanjing, China; ^3^Jiangsu Co-innovation Center for Prevention and Control of Important Animal Infectious Diseases and Zoonoses, Yangzhou, China; ^4^Jiangsu Key Laboratory for Food Quality and Safety-State Key Laboratory Cultivation Base, Ministry of Science and Technology, Nanjing, China

**Keywords:** c-di-GMP, PRV inactivated vaccine, long-term humoral immunity, T follicular helper (Tfh) cells, germinal center (GC) B cell

## Abstract

Cyclic dimeric guanosine monophosphate (c-di-GMP) is a bacterial second messenger with immunomodulatory activities in mice, suggesting potential applications as a vaccine immunopotentiator or therapeutic agent. In this study, we evaluated the efficacy of c-di-GMP as an immunopotentiator for pseudorabies virus (PRV) inactivated vaccine in a murine model. We found that c-di-GMP improved the humoral and cellular immune responses induced by PRV inactivated vaccine and its effects on immunity reached the level comparable to that of a live attenuated vaccine. Furthermore, c-di-GMP enhanced the murine antibody response against the viral glycoprotein gB up to 120 days after immunization. The c-di-GMP–adjuvanted PRV inactivated vaccine induced long-term humoral immunity by promoting a potent T follicular helper cell response, which is known to directly control the magnitude of the germinal center B cell response. Furthermore, the c-di-GMP enhanced the response of bone marrow plasma cells and upregulated the expression of *Bcl-2* and *Mcl-1*, which have been identified as anti-apoptotic regulatory genes of germinal center and memory B cells. Our findings open a new avenue for improving the immune efficacy of PRV inactivated vaccines.

## 1 Introduction

In the 50-plus years since cyclic dimeric guanosine monophosphate (c-di-GMP) was discovered, cyclic dinucleotides (CDNs) have emerged as highly versatile second messengers that control a variety of important biological processes, including biofilm development, motility, cell cycle, cell shape, and pathogenicity in bacteria, and the innate immune response in mammalian cells (1–3). CDNs identified in bacteria, which include c-di-GMP, cyclic-di-AMP (c-di-AMP), and c-GMP-AMP (c-GAMP), play important roles in virulence and the innate immune response (4, 5). It has been demonstrated that c-di-GMP induces the release of type I interferon and cytokines to activate the innate immune response by directly binding to the Stimulator of Interferon Gene (STING) protein sensor. Because the STING signaling pathway is a major player in initiating immune responses, these CDNs are believed to act as immunomodulators and mucosal adjuvants that effectively enhance antigen uptake and selectively activate pinocytosis-efficient cells *in vivo* (6, 7). Recent studies indicate that c-di-GMP increases the expression MIG/CXCL9 (a chemoattractant for activated T cells), suggesting possible antitumor activity and inhibiting basal and growth factor-induced proliferation of human colon carcinoma cells (8, 9). A proposed mechanism for the immunopotentiator properties of c-di-GMP is that STING ligation increases the production of type I interferons, which drives the adaptive immune response (10).

Pseudorabies virus (PRV), also known as Aujeszky’s disease, causes serious disease in pigs and other animals. Attenuated live or inactivated vaccines are widely used to control PRV (11, 12). The Bartha-K61 strain is a safe and effective attenuated vaccine against PRV and has played an important role in controlling and eradicating pseudorabies. However, the Bartha-K61 vaccine has not provided full protection against the prevalent PRV variants that emerged recently (13–15).

We hypothesized that effector T cell production of the Bartha-K61 vaccine could be enhanced by adding an immunopotentiator. Hence, in this study, we aimed to raise the immune efficacy of the Bartha-61 PRV inactivated vaccine to the immunity level of an attenuated live vaccine by adding c-di-GMP as an immunopotentiator in mice. The c-di-GMP PRV inactivated vaccine-mediated a long-term humoral response by inducing strong production of T follicular helper (Tfh) cells and germinal center (GC) B cells. Importantly, the c-di-GMP also induced Th1 and Th2 cell responses.

## 2. Materials and Methods

### 2.1 Cell and Viral Culture, and Immunopotentiator and Vaccine Preparation

Swine testis (ST) cells were obtained from the Institute of Veterinary Immunology & Engineering, Jiangsu Academy of Agricultural Sciences (Nanjing, China), and maintained in high-glucose Dulbecco’s modified Eagle’s medium (DMEM) supplemented with heat-inactivated 10% fetal bovine serum (FBS, HyClone, Thermo Scientific, USA), penicillin (100 U/mL) and streptomycin (100 µg/mL), at 37°C in a humidified atmosphere containing 5% CO_2_. The PRV Bartha-K61 strain was also provided by the Institute of Veterinary Immunology & Engineering and was amplified in ST cells. PRV Bartha-K61 titers were determined to be 10^8.0^ tissue culture infectious dose 50% (TCID_50_)/mL using the Reed-Muench assay. PRV virus was inactivated by adding methyl aldehyde (1:2000 v/v) (13, 14).

C-di-GMP (MedChemExpress, China) immunopotentiator was prepared as a water-in-oil emulsion formulation. Briefly, c-di-GMP (100 mg/mL)was dissolved in sterile water as the aqueous phase, mixed with inactivated PRV, and then mixed thoroughly with mineral adjuvant ISA 206 (Seppic, France).

PRV live virus (hereafter referred to as LV) was prepared by diluting live virus with DMEM to 1×10^6.0^ TCID_50_/mL, then emulsifying with an equal volume of ISA 206. PRV inactivated virus vaccine (KV) was prepared by diluting inactivated virus with DMEM and emulsified as above. To prepare PRV inactivated virus + c-di-GMP vaccine (KV-c-di-GMP), inactivated virus was diluted with DMEM as above, mixed with 100 µg c-di-GMP (100 µg/1mL), then emulsified with an equal volume of ISA 206. A blank control vaccine was prepared by emulsifying phosphate-buffered saline (PBS) with an equal ISA 206.

### 2.2 Animals and Experimental Design

Five-week-old BALB/c female mice were purchased from Yang Zhou University (Yangzhou, Jiangsu, China). The study and protocol were approved by the Jiangsu Academy of Agricultural Sciences Experimental Animal Ethics Committee of the Science and Technology Agency of Jiangsu Province (approval ID NKYVET 2015-0066). All animal studies were performed according to the guidelines of Jiangsu Province Animal Regulations (Government Decree No. 45). Eighty mice were randomly divided into four groups of 20 mice each. Each group was subcutaneously vaccinated in the neck with one of the vaccines mentioned above (100 µL per mouse).

### 2.3 Detection of PRV Glycoprotein B (gB) Antibodies in Serum

Serum samples from individual mice were collected at 7, 14, 28, 56, 90, and 120 days post-immunization (dpi) and evaluated for gB antibodies using an Aujeszky gB Antibody Test Kit (BioChek, Holland) following the manufacturer’s instructions. Briefly, 100 µL of 1:50 dilutions of serum samples were added to the appropriate wells and incubated at room temperature for 30 min. After four washes, plates were incubated with 100 µL of 1:5000 dilutions of sheep anti-mouse IgG/horseradish peroxidase for 30 min at room temperature. Plates were washed four times, 100 µL prepared substrate reagent was added to each well, then incubated for 15 min at room temperature in the dark. Finally, 50 µL stop solution was added to each well, and absorbance at 405 nm was measured within 30 min.

### 2.4 Flow Cytometric Analysis

To evaluate the frequencies of the CD3^+^CD4^+^ and CD3^+^CD8^+^ subsets of T cells induced by vaccination, samples of approximately 1×10^6^ cells/well were prepared from pools of superficial cervical lymph nodes (LNs) excised from three mice/group. Cells were incubated with Fc blocking reagent (Miltenyi Biotec, USA) in PBS plus 1% FBS (HyClone, Thermo Scientific, USA) for 10 min at 4°C, then stained with the following monoclonal antibodies (mAbs): anti-CD3 FITC, anti-CD4 PerCP and anti-CD8 PE (BD Biosciences, USA). Fluorescence-activated cell sorting (FACS) controls (1×10^6^ cells) included unstained cells or only stained with anti-CD3 FITC, anti-CD4 PerCP, or anti-CD8 PE, and appropriate isotype controls.

To characterize Tfh cell responses, superficial cervical LN single-cell suspensions were prepared by homogenization and then incubated with Fc blocking reagent in PBS plus 1% FBS for 10 min at 4°C. Approximately 1×10^6^ LN cells were stained for 30 min at 4°C with the following mAbs: anti-CD4 PerCP, anti-CXCR5 PE, and anti-ICOS Alexa648 (BD Biosciences).

To characterize GC B cell responses, superficial cervical LN single-cell suspensions were prepared by homogenization and then incubated with Fc blocking reagent in PBS plus 1% FBS for 10 min at 4°C. Approximately 1×10^6^ LN cells were stained for 30 min at 4°C with the following mAbs: anti-B220 APC (Miltenyi Biotec), and anti-GL-7 PE and anti-CD38 PE-Cyanine7 (eBioscience, USA).

For the characterization of bone marrow (BM) plasma cells (PCs), BM single-cell suspensions were generated. Cell suspensions were incubated with Fc blocking reagent in PBS plus 1% FBS for 10 min at 4°C. Approximately 1×10^6^ cells were stained for 30 min at 4°C with the following mAbs: anti-B220 APC and anti-CD138 PE (BD Biosciences).

Cells were analyzed with a BD Accuri C6 (BD Biosciences). Data analyses were performed using FlowJo version 7.6.1 software.

### 2.5 Real-Time Quantitative PCR

The expression levels of eight genes (*Bcl-6, Il-21, BCMA, Irf4, Bach2, Pax5, Bcl-2, Mcl-1*) were measured by real-time RT-PCR following vaccination of the mice with LV, KV, KV-C-di-GMP and PBS (three mice/group). TRIzol-extracted RNA from lymphocytes (1×10^6^) from superficial cervical LNs was used for reverse transcriptase (RT)-PCR with a Prime ScriptTM II Strand cDNA Synthesis kit (Takara, Dalian, China) to the manufacturer’s protocol. Real-time RT-PCR was performed for each cDNA sample in duplicate using Bright Green 2× qPCR Master Mix in a Roche Light Cycler^®^ 480 (Roche, Basel, Switzerland) with the following thermal cycling program: an initial 10 min at 95°C, followed by 40 cycles of 15 seconds at 95°C and 1 minute at 60°C. The expression level of *β-actin* was used as the internal control, and relative gene expression was measured. Primer sequences are shown in **Table 1**.

**Table 1:** Primer sequences used for real-time PCR.

Gene	forward primer (5’-3’)	backward primer (5’-3’)
Bcl-6	CCAACCTGAAGACCCACACTC	GCGCAGATGGCTCTTCAGAGTC
IL-21	TGAAAGCCTGTGGAAGTGCAAACC	AGCAGATTCATCACAGGACACCCA
BCMA	ATCTTCTTGGGGCTGACCTT	CTTTGAGGCTGGTCCTTCAG
IRF4	CCGAGGCACAGAATTGGACT	CCGTCCTTTGAATTTCGCCA
Pax5	AAGGCCATCACCATCTTCCA	TCACGCCCATCACAAACATG
Bach2	ATAACCCAGCCCTTCTCCTACC	GGCCTACGTGGTCTACATTTCC
Bcl-2	GGACTTGAAGTGCCATTGGTA	GTTATCATACCCTGTTCTCCCG
Mcl-1	TATTTCTTTCGGTGCCTTTGTG	AGTCCCGTTTCGTCCTTACA
β-actin	CACTGCCGCATCCTCTTCCTCCC	CAATAGTGATGACCTGGCCGT

### 2.6 Cytokine Quantification

To quantify relative levels of cytokines induced by vaccination, samples of approximately 1×10^6^ cells/well were prepared from pools of spleen tissue (28 dpi) excised from three mice/groups. Cells were stimulated with PRV (5×10^5^ TCID_50_/mL), or medium, overnight at 37°C, in the presence of 5% CO_2_. Supernatant samples were collected and evaluated for expression of four cytokines (interferon [IFN]-γ, interleukin [IL]-2, IL-4, IL-6) using commercially available ELISA kits (Angle Gene Technologies, Nanjing, China).

### 2.7 Statistical Analysis

Statistical analysis was performed using GraphPad Prism version 5 (GraphPad Software, San Diego, CA, USA). Statistical analyses were performed by one-way analysis of variance followed by Tukey’s t-test and Student’s t-test. Results are expressed as the mean ± standard deviation. A *P*-value of < 0.05 was considered to be statistical significance; *P*-values of < 0.01 and < 0.001 indicated greater degrees of significance.

## 3. Results

### 3.1 T Cell Response Induced by c-di-GMP in Mice

**Figure 1:**
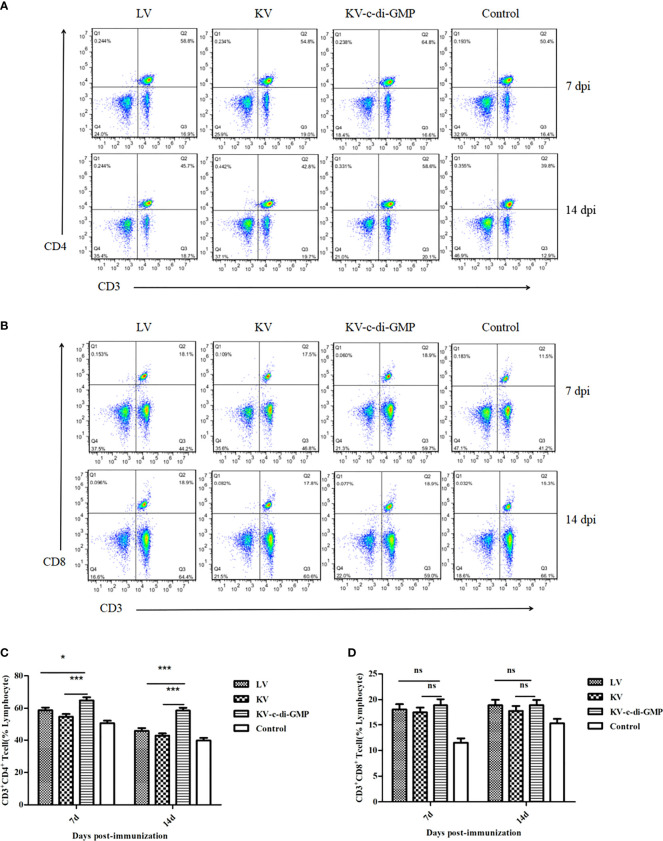
Ratios of CD3^+^CD4^+^ and CD3^+^CD8^+^ lymphocyte subsets in the vaccine and control groups at 7 and 14 dpi of mice. FACS plots of **(A)** CD3^+^CD4^+^ T cells and **(B)** CD3^+^CD8^+^ T cells are representative of one of three independent experiments. Mean frequencies ± standard deviation of **(C)** CD3^+^CD4^+^ T cells and **(D)** CD3^+^CD8^+^ T cells. *P < 0.05; ***P < 0.001; ns, no significant difference.

To evaluate the efficacy of c-di-GMP to serve as an immunopotentiator to PRV inactivated vaccine, BALB/c mice were immunized with KV vaccine with or without c-di-GMP and compared with mice immunized with LV or a PBS control. All mice tolerated the vaccine formulations with no adverse effects. To first assess the ability of c-di-GMP to induce T cell responses, subset of lymphocytes from LNs collected from all groups at 7, and 14 dpi were analyzed using FACS. At both time points, the percentages of CD3^+^CD4^+^ lymphocytes in the KV-c-di-GMP vaccine group were significantly higher than those receiving the LV, KV, or control vaccines (**Figures 1A, C**). By contrast, there were no significant differences between the vaccine groups in the percentages of CD3^+^CD8^+^ lymphocytes at either 7 or 14 dpi (**Figures 1B, D**). These results indicated that CD4+ and CD8+ T cell responses elicited by a PRV inactivated vaccine with c-di-GMP were higher and comparable, respectively, to responses elicited by a live and inactivated virus vaccination.

### 3.2 PRV-Specific gB Antibody Responses of the Vaccine Groups

**Figure 2:**
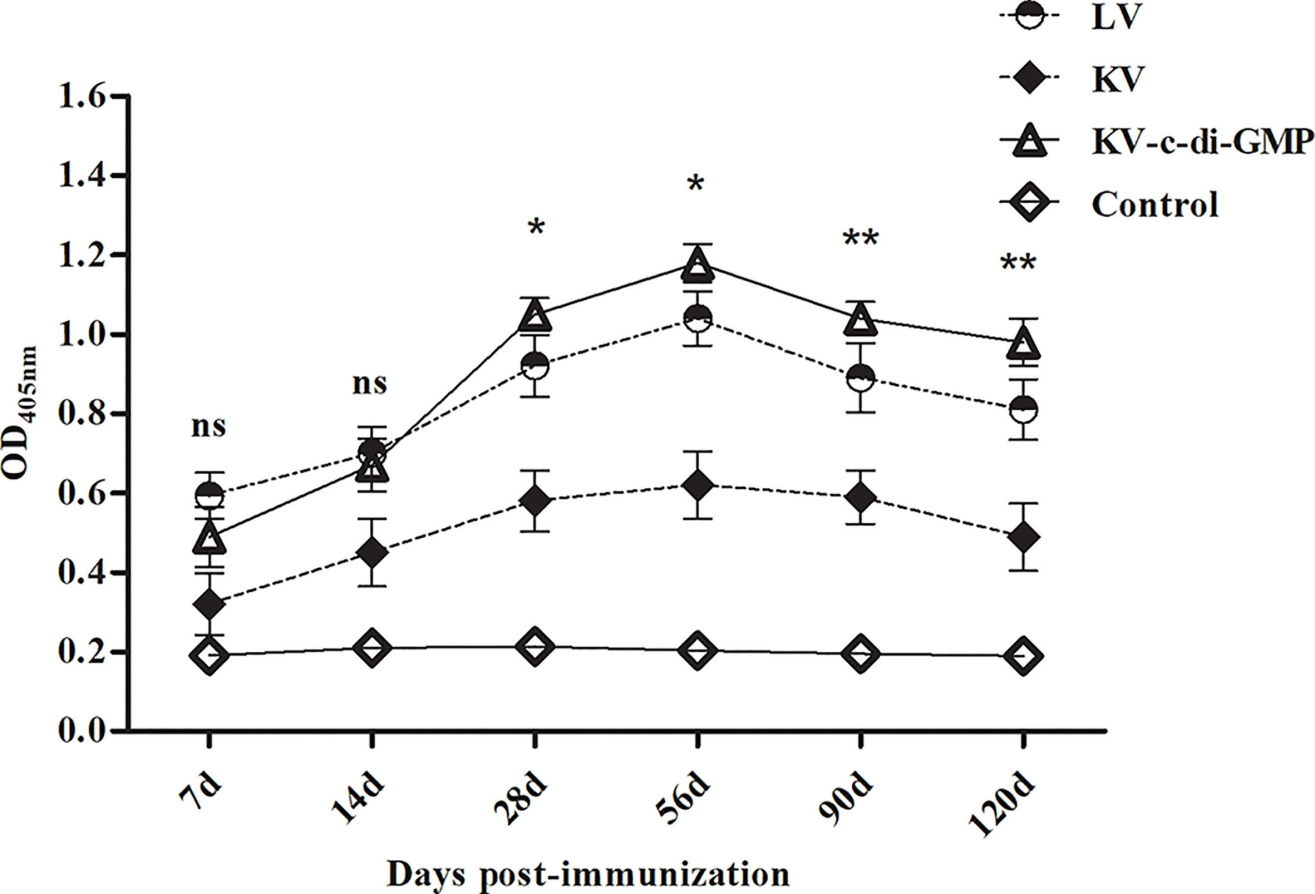
PRV-specific gB antibody responses induced by the LV, KV, and KV-C-di-GMP vaccines and PBS control in mice. Serum samples were collected at 14, 28, 56, 90, and 120 dpi. Data are expressed as the mean ± standard deviation. *P < 0.05; **P < 0.01; ns, no significant difference.

We next examined the capacity of KV with c-di-GMP to augment humoral responses. PRV-specific humoral responses were examined over time in sera from mice inoculated with the LV, KV, and KV-c-di-GMP vaccines or PBS control (**Figure 2**). As expected, LV-immunized mice possessed substantially higher antibody responses than mice receiving KV at all time points examined. The inclusion of c-di-GMP resulted in substantial augmentation of these responses. There were significant differences in the PRV-specific gB antibody response between the KV-c-di-GMP and KV groups at each sampling point and between the KV-c-di-GMP and LV groups at 28, 56, 90, and 120 dpi. Lower gB antibody responses were detected in the PBS control group at each sampling point. Collectively, we found that c-di-GMP was sufficient to raise antibody responses of a KV vaccine to levels equal to or exceeding those detected for LV-immunized mice.

### 3.3 Tfh Cell Response Induced by c-di-GMP

**Figure 3:**
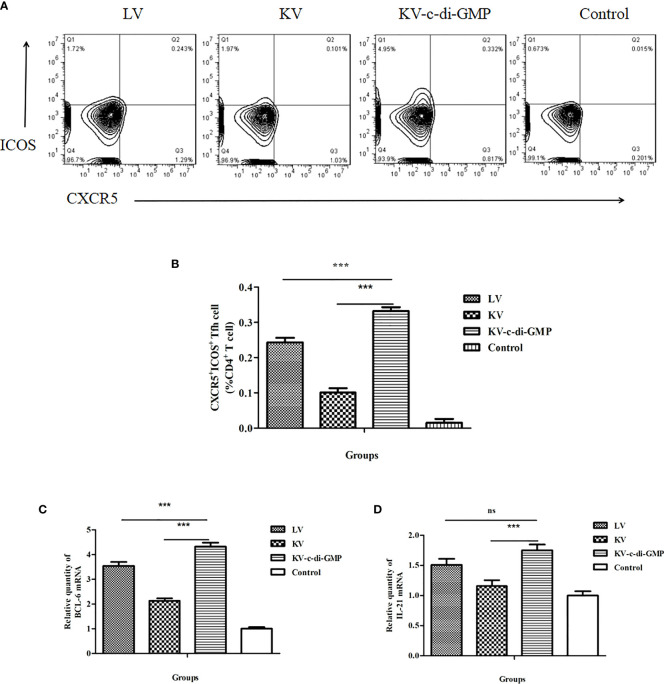
Tfh cell responses in mice induced by the LV, KV, and KV-c-di-GMP vaccines and PBS control at 28 dpi. **(A)** FACS analysis of CXCR5^+^ICOS^+^ Tfh cells. **(B)** Mean frequencies ± standard deviation of CXCR5^+^ICOS^+^ Tfh cells. Data represent three independent experiments. Pooled LNs from three mice were processed independently three times were analyzed by real-time PCR for **(C)** *Bcl-6* and **(D)** *IL-21* expression. ***P < 0.001; NS, no significant difference.

Tfh cells play an important role in GC response and formation of PCs from the BM (16). To assess the capacity of c-di-GMP to modulate these cells, percentages of CXCR5^+^ICOS^+^CD4^+^ Tfh cells in LNs at 28 dpi were analyzed by FACS in each group (**Figure 3A**). Compared with the KV and LV vaccines, KV-c-di-GMP induced production of a significantly higher percentage of Tfh cells in mice (**Figure 3B**), supporting the immunopotentiator activity of c-di-GMP.

The transcriptional repressor B-cell lymphoma (BCL)-6 protein is known to instruct naive CD4^+^ T cells to commit to a Tfh fate. Tfh cells then signal B cells that recognize cognate antigens through the surface and secreted molecules, including CD40L, inducible costimulator (ICOS), and IL-21 (17, 18). To further examine the Tfh response induced by the KV-c-di-GMP vaccine, we quantified the expression levels of *Bcl-6* and *Il-21* in the lymphocytes of LNs using real-time RT-PCR. As shown in **Figure 3C**, *Bcl-6* expression was significantly higher in the group of mice vaccinated with KV-c-di-GMP than in the group receiving KV or LV. The expression levels of *Il-21* were significantly higher in the KV-c-di-GMP group compared with those in the KV group (*P* < 0.001), but there was no significant difference between the KV-c-di-GMP and LV groups (**Figure 3D**).

### 3.4 Influence of c-di-GMP on GC Response

**Figure 4:**
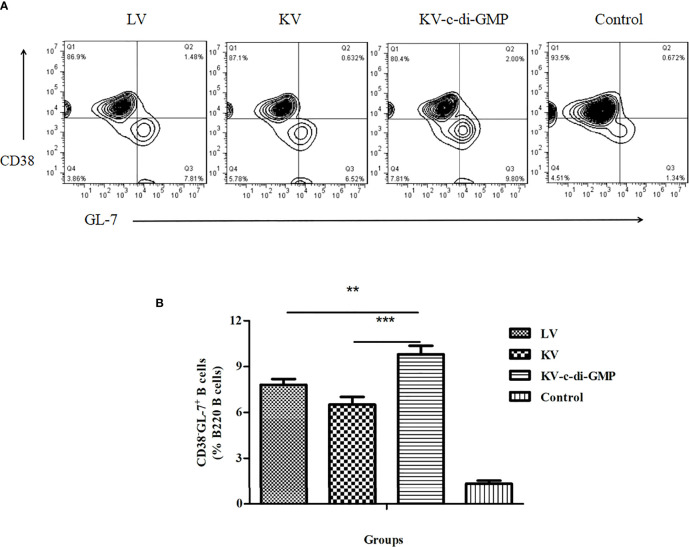
Frequencies of GC B cells in the LNs of the four groups at 28 dpi. **(A)** FACS analysis of B220^+^CD38^-^GL-7^+^ B cells. **(B)** Mean frequencies ± standard deviation of B220^+^CD38^-^GL-7^+^ B cells. Data represent three independent experiments. Pooled LNs from three mice were processed independently three times. **P < 0.01; ***P < 0.001.

Because GCs are the sites responsible for the activation and maturation of B cells, we also analyzed the B cell response in terms of the percentage of B220^+^GL-7^+^CD38^-^ cells in the LNs at 28 dpi (**Figure 4A**). There was a significantly higher B cell response in the KV-c-di-GMP group than in the KV and LV groups (*P* < 0.001, *P* < 0.01; **Figure 4B**), further supporting c-di-GMP immunopotentiator capacity when adjuvanted to the KV vaccine.

### 3.5 Enhanced Differentiation of BM PCs in Mice

**Figure 5:**
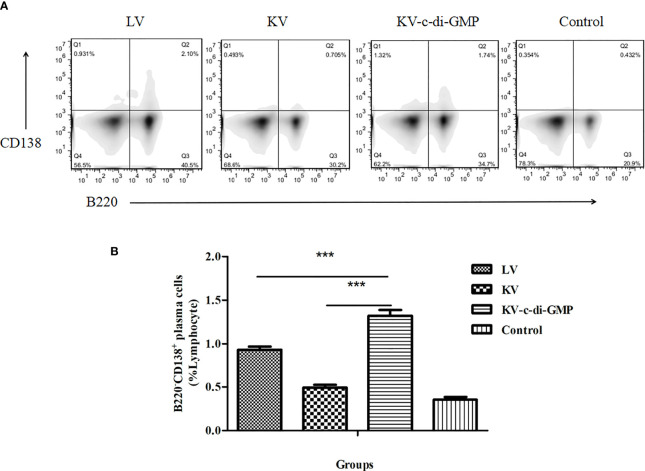
Frequencies of B220^−^CD138^+^ BM PCs at 28 dpi. **(A)** FACS analysis of B220^−^CD138^+^ in BM PCs. **(B)** Mean frequencies ± standard deviation of B220^−^CD138^+^ BM PCs. Data represent three independent experiments. BM PCs from three mice were processed independently three times. ***P < 0.001.

Long-lived humoral immunity is manifested by the ability of BM PCs to survive for extended periods. FACS analysis of the differentiation of BM PCs revealed a significantly higher percentage of B220^−^CD138^+^ BM PCs in the KV-c-di-GMP group compared with the percentages in the KV and LV groups (*P* < 0.001, *P* < 0.001; **Figure 5**).

### 3.6 Expression of Transcription Factors Important for B Cells

**Figure 6:**
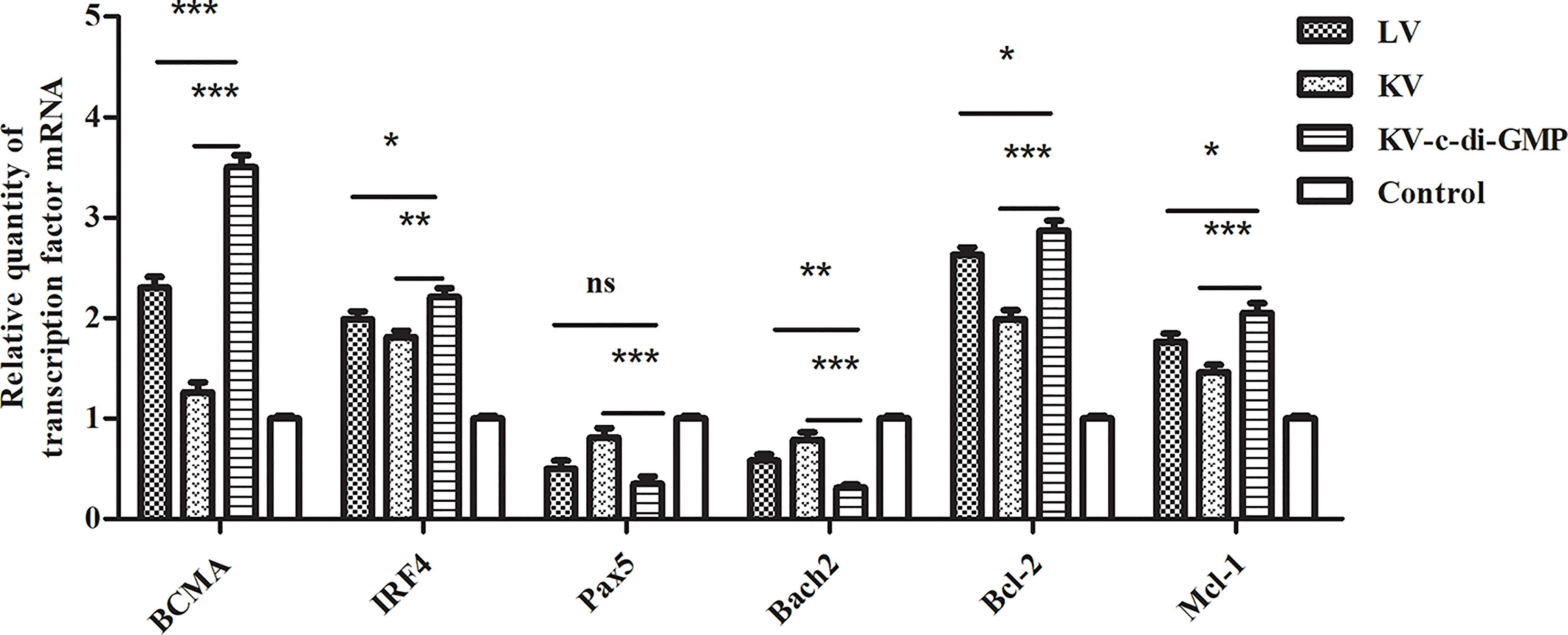
Real-time RT-PCR showing the relative expression of transcription factor genes in lymphocytes from LNs at 28 dpi. The relative gene expression was calculated by 2 -ΔΔCt. ΔΔCt = (Ct [target gene] -Ct [reference gene])_experimental group_ - (Ct [target gene] -Ct [reference gene])_control group_. Data are expressed as mean ± standard deviation from three mice/group and represent three independent experiments. Pooled LNs from three mice were processed independently three times. *P < 0.05; **P < 0.01; ***P < 0.001; ns, no significant difference.

Transcription factors including BCMA, Pax5, Bach2, IRF4, Bcl-2, and Mcl-1 have recently been shown to play integral roles in developing mature B cells and the differentiation or survival of activated B cells (19, 20). Using real-time RT-PCR, we assessed the effects of the vaccines on relative mRNA expression levels of these six transcription factors in lymphocytes from LNs at 28 dpi (**Figure 6**). Compared with those in the PBS control group, the expression of *BCMA* and *Irf4* were upregulated, while *Pax5* and *Bach2* were downregulated, in all three vaccine groups, with significant differences between the KV-c-di-GMP group and the KV and LV groups. Anti-apoptotic genes *Bcl-2* and *Mcl-1* were also significantly upregulated in the KV-c-di-GMP group compared with those in the KV and LV groups, indicating that inclusion of c-di-GMP to a KV vaccine was capable of supporting the production of transcription factors at or higher than those observed with an LV vaccination approach.

### 3.7 Induction of Cytokine Expression by c-di-GMP

**Figure 7:**
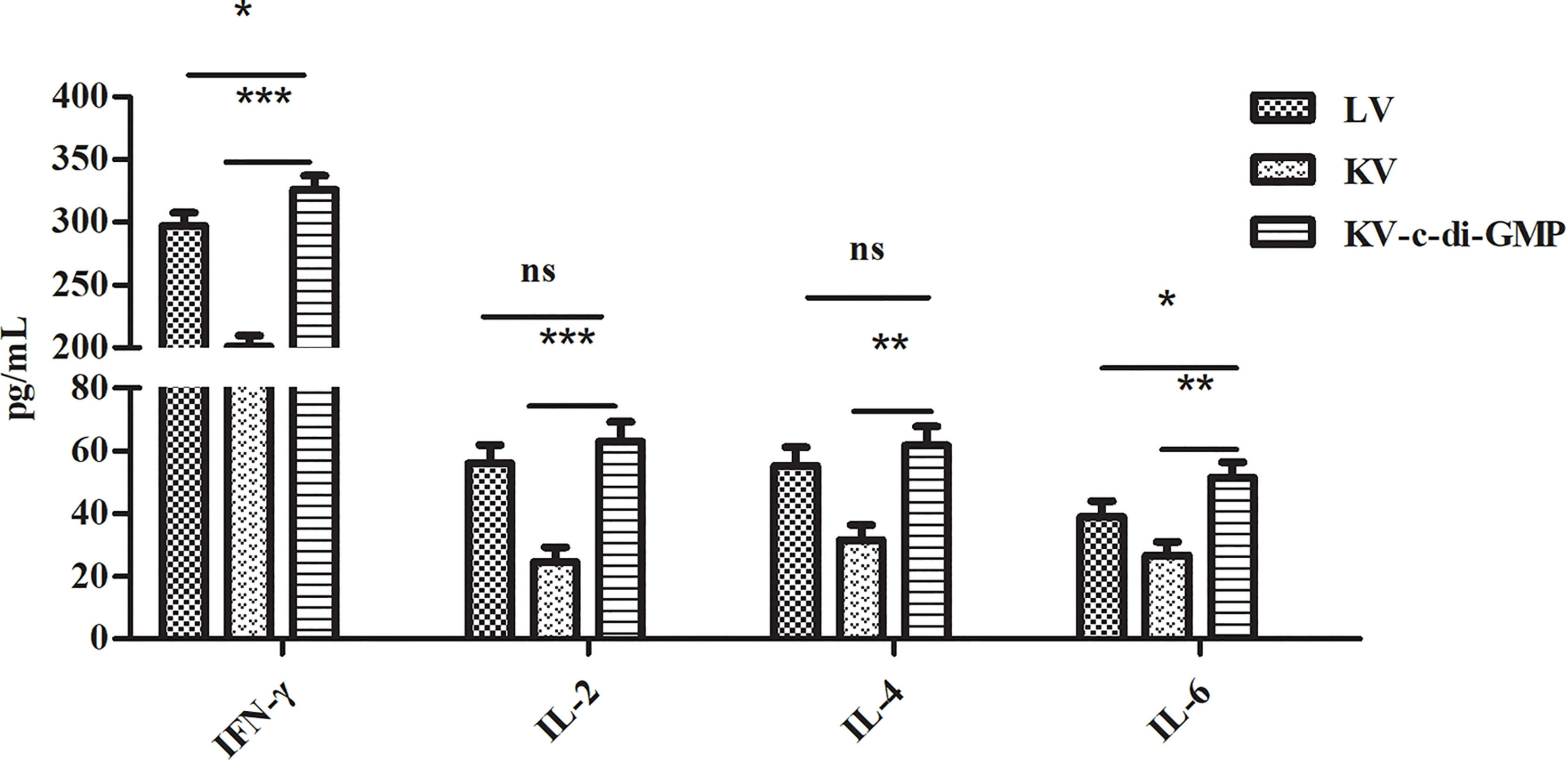
Cytokine induction by the three vaccines in spleen cells isolated at 28 dpi and re-stimulated with PRV *in vitro*. Levels of IFN-γ, IL-2, IL-4, and IL-6 by ELISA are expressed as mean ± standard deviation from three mice/group. Data represent three independent experiments. *P < 0.05; **P < 0.01; ***P < 0.001; ns, no significant difference.

Enhancement of the cellular immune response can improve the ability of animals to ward off viral infection. We used ELISA to examine the vaccine-mediated induction of cytokines in spleen cells isolated at 28 dpi and re-stimulated with PRV *in vitro* (**Figure 7**). The highest levels of all four cytokines were observed in the KV-c-di-GMP group. Specifically, the levels of IFN-γ in the KV-c-di-GMP, KV, and LV groups were 326 ± 10.98 pg/mL, 201 ± 8.56 pg/mL, and 297 ± 10.62 pg/mL, respectively. The levels of IL-2 in the KV-c-di-GMP, KV, and LV groups were 63 ± 6.2 pg/mL, 24.6 ± 4.8 pg/mL, and 56.2 ± 5.7 pg/mL, respectively. The levels of IL-4 in the KV-c-di-GMP group (61.8 ± 5.97 pg/mL) were significantly higher than those in the KV group (31.6 ± 4.83 pg/mL), but not the LV group (55.3 ± 5.87 pg/mL). The levels of IL-6 in the KV-c-di-GMP, KV, and LV groups were 51.6 ± 4.87 pg/mL, 26.8 ± 4.32 pg/mL, and 39.0 ± 4.98 pg/mL, respectively. Collectively, these data support the efficacy of c-di-GMP to serve as an immunopotentiator to the KV vaccine to elicit Th1 and Th2 immune responses comparable to or exceeding those of an LV vaccine.

## 4 Discussion

The immunomodulatory activity of any given vaccination strategy is determined by the relevant antigenic components in the vaccine formulation and the complement of a suitable immunopotentiator (21, 22). Toll-like receptor agonists have been widely used as immunopotentiators in veterinary vaccines (23). Several studies have emphasized the immunopotentiator potential of the bacterial second messenger c-di-GMP (24, 25). A proposed mechanism for the immunopotentiator properties of c-di-GMP is that STING ligation increases the production of type I IFNs, which drives the adaptive immune response (10). This study found that c-di-GMP in a PRV inactivated vaccine confer heightened immunopotentiator activity of the vaccine.

T cells are a central component of immune defense against pathogens. CD4^+^ T helper (Th) cells are central in immune and autoimmune responses (26). Like CD8^+^ cytotoxic T cells, Th cells develop in the thymus, with specificity to recognize major histocompatibility peptide complexes on antigen-presenting cells. After activation, CD4^+^ Th cells differentiate into effector subsets of Th1 cells, which enhance the cellular immune response by producing IFN-γ and IL-2, or Th2 cells, which enhance the humoral immune response by producing IL-4, IL-6, and IL-13 (27, 28). In this study, the percentages of CD3^+^/CD4^+^ cells were significantly higher in the KV-c-di-GMP group than in the LV or KV groups, but the percentages of CD3^+^/CD8^+^ were similar in the two groups. Additionally, the levels of IFN-γ, IL-2, IL-4, and IL-6 production in the spleen were significantly higher in the KV-c-di-GMP group than in the KV group. These results indicated that c-di-GMP could boost innate and adaptive immune responses.

PRV is a 150 kb linear double-stranded DNA virus encoding more than 70 proteins. The viral glycoproteins gB, gC, and gD induce protective immune responses (29, 30). Of the three, gB reportedly induces the strongest antibody response and provides the most effective protection against virulent PRV (31). This study showed that the gB antibody levels remained higher in the KV-c-di-GMP group than in the LV or KV groups up to 120 days after immunization. Thus, c-di-GMP was an effective immunopotentiator, inducing long-term antibody immunity following immunization with the PRV inactivated vaccine.

The success of most vaccines lies in the generation of antibodies to provide protection against subsequent infection, a robust GC response that culminates in the production of long-lived antibody-secreting PCs (32, 33). The GC response is directed by a specialized subset of CD4^+^ T cells: the Tfh cells. Tfh cells provide growth and differentiation signals to GC B cells and mediate positive selection of high-affinity B cell clones in the GC (34). In our effort to further elucidate the immunological mechanism by which c-di-GMP co-administered with KV induced long-term antibody immunity, we showed that c-di-GMP enhanced the percentage and the total number of Tfh cells and increased the expression levels of key molecules, including *Bcl-6* and *Il-21* (the functional hallmarks of GC B cells and Tfh cells) (35, 36). We also found that the KV-c-di-GMP vaccine induced significantly higher percentages of GC B cells and BM PCs than the LV or KV vaccines, suggesting that the c-di-GMP in the PRV inactivated vaccine played an important role in immune responses. Transcription factors including BCMA, IRF4, Bach2, and Pax5 are essential for PC differentiation. The levels of mRNA expression of *BCMA* and *IRF4*, which promote the generation and differentiation of plasmablasts, were upregulated in the KV-c-di-GMP group, while those of *Bach2* and *Pax5*, which antagonize PC differentiation, were downregulated in the KV-c-di-GMP group. Two anti-apoptotic genes, *Bcl-2* and *Mcl-1*, were also upregulated in the KV-c-di-GMP group.

An ideal vaccine against an infectious pathogen elicit a potent humoral immune response as well as a robust cellular immune response. Inactivated-virus vaccines perform poorly in terms of stimulating cellular immunity. This study improved both the humoral and cellular immune responses to the Bartha-K61 PRV inactivated vaccine in mice by adding c-di-GMP as an immunopotentiator. Furthermore, we deciphered the immunological mechanism of c-di-GMP in inducing long-term antibody immunity. We propose that c-di-GMP has the potential for development as an immunopotentiator to improve the efficacy of PRV inactivated vaccines.

## Data Availability Statement

The original contributions presented in the study are included in the article/supplementary material. Further inquiries can be directed to the corresponding authors.

## Ethics Statement

The animal study was reviewed and approved by Jiangsu Academy of Agricultural Sciences Experimental Animal Ethics Committee, Science and Technology Agency of Jiangsu Province (approval number: NKYVET 2015-0066).

## Author Contributions

LH, QZ, JC, and JH designed the experiments. A sampling of serum, LNs, and bone marrow were mainly performed by LH, LD, XY, and YYZ. LH, YPZ, and HC analyzed the results with guidance from QZ and JH and drafted the manuscript. All authors took part in the discussion and interpretation of the results. All authors read, advised, and approved the final manuscript.

## Funding

This work was supported by the National Natural Sciences Foundation of China (32102690), Jiangsu Agricultural Science and Technology Innovation Fund (CX [20]3096), Jiangsu Agricultural Science and Technology Innovation Fund (CX [21]3135).

## Conflict of Interest

The authors declare that the research was conducted in the absence of any commercial or financial relationships that could be construed as a potential conflict of interest.

## Publisher’s Note

All claims expressed in this article are solely those of the authors and do not necessarily represent those of their affiliated organizations, or those of the publisher, the editors and the reviewers. Any product that may be evaluated in this article, or claim that may be made by its manufacturer, is not guaranteed or endorsed by the publisher.
